# Kratom, an Emerging Drug of Abuse, Raises Prolactin and Causes Secondary Hypogonadism: Case Report

**DOI:** 10.1177/2324709618765022

**Published:** 2018-03-16

**Authors:** Lauren LaBryer, Rohan Sharma, Kaustubh Suresh Chaudhari, Mitali Talsania, Robert Hal Scofield

**Affiliations:** 1 University of Oklahoma Health Sciences Center, Oklahoma City, OK, USA; 2 Oklahoma Medical Research Foundation, Oklahoma City, OK, USA; 3 Veterans Affairs Medical Center, Oklahoma City, OK, USA

**Keywords:** kratom, hypogonadism, prolactin, hyperprolactinemia

## Abstract

*Background.* Kratom is a drug derived from the leaves of the tree *Mitragyna speciose*, which is native to southern Thailand. The route of administration is oral. Kratom has become increasingly available in the United States. The active ingredients in the drug bind the opioid mu-receptor; therefore, kratom has similar physiological effects as mu-opioids. Elevated prolactin is a common medical condition frequently caused by a variety drugs, including opioids. *Case Report.* A 42-year-old man presented with poor energy and low libido. He had mildly elevated serum prolactin with hypogonadotropic hypogonadism as evidenced by low serum testosterone with luteinizing hormone and follicle-stimulating hormone in the normal range. At his initial visit, he reported no use of any recreational or therapeutic drug. Two months later when seen in follow-up, both the testosterone and prolactin levels had returned to normal. At that visit he reported frequent use of kratom, which he had discontinued a few days after the first visit. *Discussion*. Kratom is now widely available in health food stores and online and is considered an emerging drug of abuse. At present kratom is legal in the United States, but recently the Drug Enforcement Administration served noticed of its intention of making kratom a Schedule I drug. A number of adverse events or side effects have been reported, but this is the first report of hyperprolactinemia as the result of ingestion of kratom.

*Mitragyna speciose*, or kratom, is a tropical tree native to Southern Thailand. The leaves of kratom, either chewed or brewed as a drink, are used as a stimulant and for chronic pain, opioid withdrawal, and alcohol dependence in Thailand. Although possession of kratom is prohibited under law in Thailand and Malaysia, kratom is now emerging as a recreational drug of abuse in the Western world. It has become widely available from natural product stores and via the Internet and is touted as a safe and legal psychoactive agent.^1^ Kratom is regulated in certain states like Alabama but not controlled under the Federal Controlled Substances Act. Furthermore, its use has increased in the United States since 2009.^2^ The principle active ingredients in kratom are mitragynine and 7-hydroxymitragynine. These alkaloid compounds bind the opioid mu-receptor and produce both excitation and sedation effects.^1,3-5^ Reported medical consequences of ingestion of kratom are cholestasis,^6,7^ seizures,^8^ hypothyroidism,^9^ psychosis,^8^ as well as sedation, loss of appetite, constipation, respiratory depression, and other opioid-like side effects. Chronic kratom intake can be followed by opioid-like withdrawal symptoms on cessation of drug use. Physical withdrawal symptoms include tremor, sweating, muscle spasms, hot flashes, watery eyes and nose, decreased appetite, and diarrhea. Psychological withdrawal symptoms like anxiety, restlessness, anger sadness, and craving have also been reported.^5,10,11^ There have been reports of coma^12^ and even fatality related to kratom overdose.^13-15^ Kratom and its metabolites are not detected by a typical drug screen and require special testing.

We report a 42-year-old white man who presented to his primary care physician with complaints of low energy and poor libido. He was found to have a mildly elevated serum prolactin at 24 ng/mL (upper limit = 12 ng/mL) and hypogonadotropic hypogonadism. He was referred to endocrinology where no pituitary pathology was found. A thyroid-stimulating hormone test was normal. Free T4 was not determined. He was not taking any prescription medication known to affect prolactin level. He reported no use of androgenic steroids or recreational drugs at his initial visit. Two months later the prolactin was only 6 ng/mL, the total testosterone was back in the normal range, and the symptoms had resolved (see Table 1). At this follow-up visit he reported use of kratom at the time of the initial laboratory test but had stopped about 2 months prior to the follow-up laboratory tests, which showed a normal serum testosterone.

**Table 1.** Prolactin, Testosterone, LH, and FSH in a Patient While Taking as Well as After Stopping Kratom.

	On Kratom	After Stopping	Reference Range
Prolactin	24	6	2.6-13 ng/mL
Testosterone	8.4	14.6	8.7-25.1 pg/mL
LH	6.8	ND	1-9 mIU/mL
FSH	7.7	ND	1-19 mIU/mL
TSH	0.77	ND	0.4-4.5 mIU/mL

Abbreviations: LH, luteinizing hormone; FSH, follicle-stimulating hormone; ND, not done.

## Discussion

Kratom is a drug little known in the Western world but has become increasingly available in the United States.^2^ Use of this substance is addictive and stoppage results in withdrawal symptoms.^3,11^ Use of this plant product is associated with a few medical problems, as discussed above. At present kratom is legal in the United States, excepting a few select states. But a Notice of Intent by the US Drug Enforcement Agency to make the active ingredients, mitragynine and 7-hydroxymitragynine, Schedule I^16^ has recently been withdrawn.

Hyperprolactinemia is a common problem in both men and women.^17^ Common etiologies include prolactin-secreting pituitary adenomas (both microadenoma and macroadenoma), hypothyroidism, and drug effect. There are several classes of drug that induce hyperprolactinemia, which include neuroleptics, atypical antipsychotics, selective serotonin reuptake inhibitors, antiemetics, calcium channel blockers, opiates, H_2_ blockers, and others. Most of these drugs stimulate prolactin secretion because of their antidopaminergic effects on tuberoinfundibular dopamine pathway in the central nervous system.

Hyperprolactinemia usually manifests with symptoms of hypogonadism.^18^ Women report clinical symptoms earlier than men mostly because of changes in menstrual cycles. Common symptoms in women are amenorrhea, oligomenorrhea, galactorrhea, infertility, and decreased libido. Men tend to report late, as there is no reliable indicator like menses. Male patients usually present with loss of libido, infertility, impotence, gynecomastia, and rarely galactorrhea. There might be associated symptoms of headache or vision loss if the etiology is pituitary gland adenoma.

Our patient’s serum prolactin was in the range associated with drug effect or hypothyroidism, which was not present. Drugs that potentially raise prolactin include dopamine agonists and serotonin reuptake inhibitors, antipsychotics, as well as opioids. However, he was taking none of these medications but instead was using kratom. When he stopped kratom, the serum prolactin and testosterone normalized. This sequence strongly suggests kratom intake was causally related to the change in serum prolactin level. Since opioids are known to produce similar effects, we hypothesize that kratom, by virtue of binding and activating opioid receptors, raises prolactin. Another possibility is an estrogenic effect of kratom but we find no report of such an effect. As recreational use of this presently legal drug increases, physicians should be aware of this previously unreported complication.
